# Pediatric Palliative Care: Communication, Decision-Making, Care, and Support Amid Complexity and Uncertainty

**DOI:** 10.3390/children13050693

**Published:** 2026-05-19

**Authors:** DonnaMaria E. Cortezzo

**Affiliations:** 1 Division of Neonatology, Connecticut Children’s, Hartford, CT 06106, USA; dcortezzo@connecticutchildrens.org; 2 Division of Pain and Palliative Care, Connecticut Children’s, Hartford, CT 06106, USA; 3 Fetal Care Center, Connecticut Children’s, Hartford, CT 06106, USA; 4 Department of Pediatrics, University of Connecticut School of Medicine, Farmington, CT 06030, USA

Pediatric palliative care (PPC) is an essential, evolving component of care for children with serious, complex, life-threatening, or life-limiting conditions. Over the past two decades, its scope has expanded beyond the historical focus on end-of-life care to encompass comprehensive, interdisciplinary support from the time of diagnosis throughout the illness course. At times, it is offered in tandem with life-sustaining and cure-directed therapies. The World Health Organization and the American Academy of Pediatrics emphasize that high-quality PPC extends beyond the management of physical symptoms to address the emotional, psychosocial, cultural, and spiritual needs of children and their families [1,2,3]. Quality care includes alleviating suffering, improving quality of life, supporting informed value-driven and shared decision-making, and fostering meaningful communication. This Special Issue, *Pediatric Palliative Care and Pain Management*, highlights the multifaceted challenges inherent in caring for children with serious illness whilst offering important insights, advancing and improving care across diverse settings.

The influence of cultural and systemic factors on PPC delivery is explored by Zhong et al. in their study that evaluates the barriers and facilitators of PPC implementation across mainland China, Hong Kong, and Taiwan [4]. They explore the ways in which cultural stigma surrounding death and dying, variability in healthcare infrastructure, and differences in policy, training, and resource allocation continue to affect the availability, accessibility, and quality of PPC services. The authors also identify important opportunities for growth, including increasing public and professional awareness, expanding education and workforce development, and fully integrating PPC into existing healthcare systems. Their findings reinforce that PPC implementation and advancement are influenced by cultural and systemic contexts, and emphasize the necessity of local adaptation and global collaboration.

An important theme associated with quality PCC delivery is skilled communication during complex discussions. In their narrative review, Benedetti et al. explore the ways in which cultural, religious, and spiritual factors shape communication with the families of children with severe neurological conditions [5]. These factors impact how information is received, interpreted, and incorporated into decision-making. Effective communication requires an understanding of the relevant medical information in addition to cultural humility, emotional insight, and adaptability. These skills are especially important in the context of prognostic uncertainty, where families explore meaning, hope, and fears as they navigate difficult decisions.

Complementing this perspective, Jaaniste et al. explore healthcare information avoidance among caregivers of children with serious illness [6]. While access to information is often assumed to be inherently empowering, families may at times intentionally avoid medical information as a coping strategy. Information preferences vary significantly over time and between individuals. Healthcare professionals should recognize families’ emotional readiness, coping capacity, and communication preferences and adapt their communication approaches to meet their evolving needs. Respecting these dynamics fosters trust and supports shared decision-making in a compassionate and individualized manner.

Decision-making and communication in the face of uncertainty are challenging and complex aspects of PPC. In their article, Cortezzo et al. present a framework for navigating complex genomic testing in critically ill neonates [7]. The authors describe the challenges healthcare professionals and families encounter when interpreting uncertain, nondiagnostic, or difficult-to-contextualize genetic testing results in the context of goals-of-care discussions. Illustrative clinical scenarios demonstrate how genomic information can significantly influence prognostic understanding, communication, and shared decision-making. The proposed “PICTURE” framework emphasizes the importance of multidisciplinary collaboration among neonatology, genetics, and palliative care teams while promoting transparent communication, acknowledgment of uncertainty, and integration of family values into medical decision-making.

While much of this Special Issue revolves around communication and decision-making, symptom management and innovative therapies, a foundational cornerstone of PPC, is also represented. Nicolosi et al. describe the use of blue light therapy for pediatric skin injuries [8]. This is a safe, well-tolerated, and non-invasive option for promoting wound healing across a range of pathologies. Established and emerging therapeutic approaches of this nature support several important goals of PPC, such as alleviating suffering, enhancing comfort, and improving quality of life. Effective symptom management can reduce emotional distress, support developmentally meaningful activities, lessen caregiver burden, and preserve a child’s sense of identity and dignity. Integrating innovative supportive therapies into comprehensive PPC models is essential for improving the quality of life of children and families facing serious illness.

The collected papers provide meaningful and timely insight into the evolving field of PPC and deepen our understanding of how communication, culture, decision-making, and symptom management intersect in this context. They also highlight the importance of understanding the experiences, values, priorities, and goals of children and families navigating serious illness. Across healthcare settings and clinical contexts, compassionate communication, interdisciplinary collaboration, and individualized care are pivotal. This reinforces the importance of the ongoing development of high-quality, equitable, and value-driven PPC.

Future research should focus on identifying interventions that improve communication, reduce disparities in access to PPC services, and support families facing uncertainty and emotionally complex decision-making. The ethical and emotional implications of prognostic uncertainty, particularly in the presence of rapidly evolving technologies, warrant further exploration. Establishing a deeper understanding of caregiver experiences, including caregiver burden, anticipatory grief, psychological distress, resilience, and long-term bereavement outcomes is equally important. Future work should also continue to examine how healthcare systems, institutional culture, and public perceptions influence the integration and accessibility of PPC across different regions and populations.

As the field of PPC continues to evolve, sustained interdisciplinary collaboration, culturally responsive care models, and thoughtful integration of emerging technologies are paramount. Efforts to normalize earlier integration PPC may help to improve healthcare professionals’, patients’, and families’ understanding of the comprehensive role of palliative care. Additionally, continued innovation in symptom management, communication strategies, and psychosocial support is essential for improving the quality of life of children living with serious illness and their families. The continued evolution of PPC should remain centered on relieving suffering, preserving dignity, fostering meaningful connection, and walking alongside children and families with compassion, honesty, and humanity through the profound uncertainty of serious illness.
